# A Comprehensive Evaluation of CAR-T Cell Gene Therapy, Tracing its Revolutionary Clinical Breakthroughs and Advancements Towards Next-Generation Engineering

**DOI:** 10.1017/erm.2026.10052

**Published:** 2026-05-15

**Authors:** Melike Aliciaslan, Ezgi Erbasan, Salih Sanlioglu

**Affiliations:** Akdeniz University: Akdeniz Universitesi, Türkiye

**Keywords:** CAR-T cell therapy, cancer gene therapy, gene editing, immunotherapy, T-cell engineering

## Abstract

**Background:**

CAR-T cell gene therapy has advanced from an experimental concept to a standard curative treatment for selected hematologic malignancies. Substantial clinical evidence has established CAR-T therapy as a cornerstone for relapsed/refractory B-cell malignancies and multiple myeloma, demonstrating durable, long-term remissions. In 2025, the U.S. Food and Drug Administration (FDA) eliminated the Risk Evaluation and Mitigation Strategy (REMS) requirement, reflecting improved clinical management of Cytokine Release Syndrome (CRS) and Immune Effector Cell-Associated Neurotoxicity Syndrome (ICANS). Yet, the widespread adoption of these therapies has unveiled a new landscape of long-term and next-generation challenges.

**Methods:**

This review critically analyzes CAR-T therapy’s clinical trajectory and the bioengineering strategies redefining its safety, scalability, and translational potential.

**Results:**

A major hurdle is therapeutic resistance driven by antigen escape, notably through alternative splicing and lineage switching. The emergence of long-term safety signals, specifically secondary T-cell malignancies, has necessitated rigorous surveillance, exemplified by the European Medicines Agency’s (EMA) mandate for lifelong patient monitoring. The therapeutic landscape is further complicated by histopathological and immunological barriers limiting the expansion and persistence of engineered cells. These include deficient T-cell trafficking, an immunosuppressive tumor microenvironment (TME), and antigen heterogeneity, which have historically constrained efficacy in solid tumors. To address these hurdles, the field is leveraging CRISPR-enhanced allogeneic platforms, cytokine-secreting armored CARs (TRUCKs), and logic-gated systems showing early clinical promise.

**Conclusion:**

Building on these developments, we hypothesize that CAR-T therapy is undergoing a paradigm shift from single-target cytotoxicity toward a multi-functional, programmable framework capable of overcoming resistance, enhancing safety, and enabling effective penetration of solid tumors.

## Introduction: The CAR-T therapy revolution in oncology

The shift in therapeutic paradigms driven by CAR-T cells necessitates a precise understanding of their underlying bioengineering. By clarifying the conceptual framework of engineered T-cell function, we provide the groundwork for evaluating the next generation of clinical innovations. Building on the clinical success of CAR-T therapy in B-cell malignancies, the field has transitioned from proof-of-concept validation to addressing complex hurdles such as long-term durability, antigen escape and limited efficacy in solid tumours. Consequently, research is shifting towards advanced synthetic biology specifically programmable and off-the-shelf allogeneic platforms aimed at enhancing persistence and bypassing the unique biological barriers of solid malignancies.

### Fundamental principles of CAR-T therapy

CAR-T therapy represents a living drug platform at the intersection of gene therapy, cell therapy and immunotherapy (Ref. 1). The therapy relies on a complex, multi-step process of *ex vivo* genetic engineering (Ref. 2). T-lymphocytes are first harvested from the patient (autologous) or a healthy donor (allogeneic) via leukapheresis. These T cells are then genetically engineered, most commonly using a lentiviral or retroviral vector, to express a synthetic CAR on their surface. The engineered cells are expanded to billions within a bioreactor system. To prepare for adoptive transfer, the patient receives a lymphodepleting conditioning regimen designed to optimize the host environment for T-cell expansion. Following reinfusion, CAR-expressing T cells recognize tumour-associated antigens in an MHC-independent manner and initiate targeted cytotoxic responses upon antigen encounter, thereby redirecting endogenous T-cell effector functions towards malignant cells (Ref. 3).

### Generations of CARs and their evolutionary history

**Figure 1.**
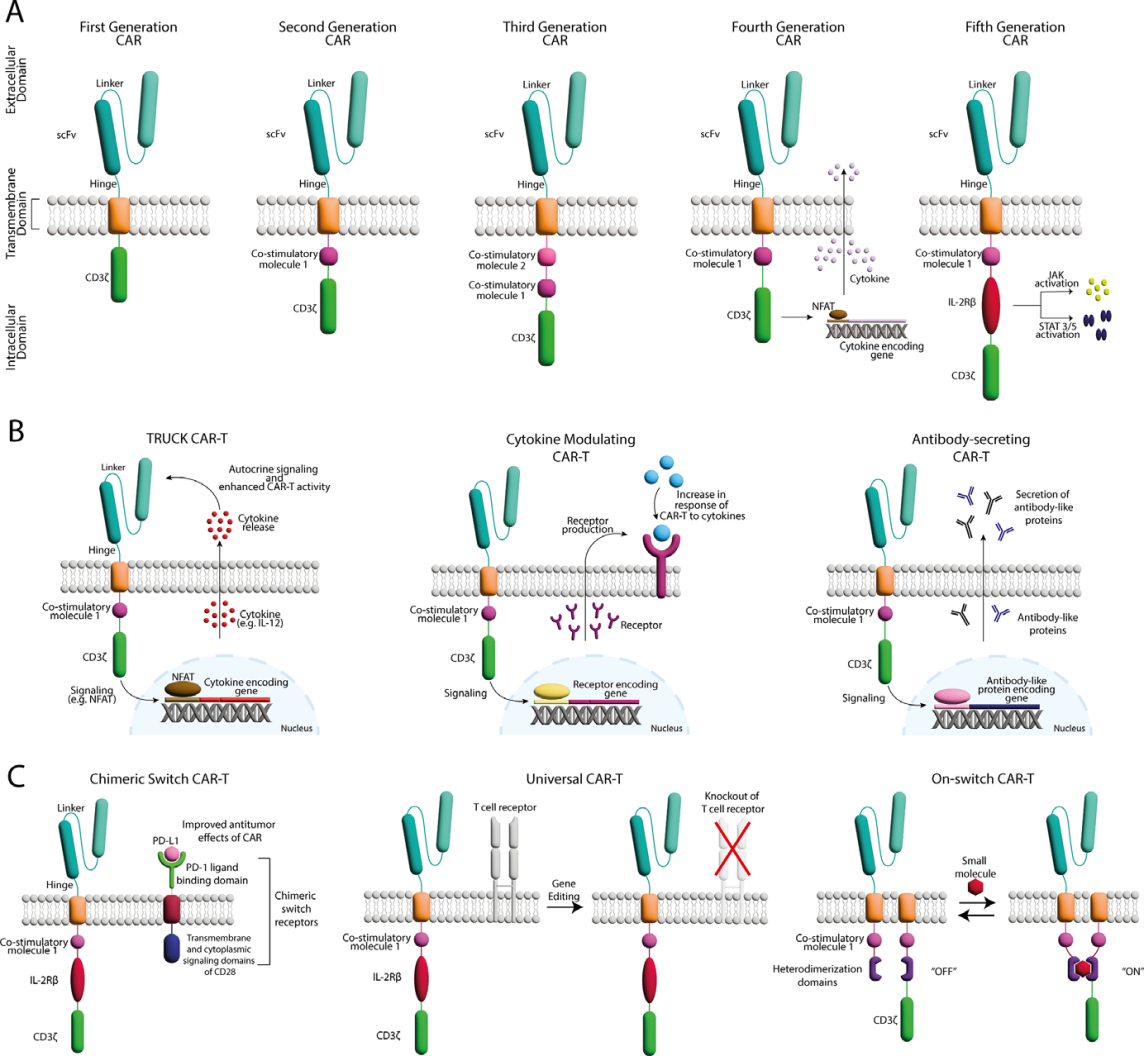
Progressive engineering of CAR constructs and advanced armoured CAR-T strategies (10, 49, 119). **A.** Overview of the structural and functional evolution of CAR design. First-generation CARs contain only the CD3ζ activation domain, exhibiting limited proliferation and persistence. Second-generation CARs incorporate a single costimulatory domain (typically CD28 or 4-1BB), forming the backbone of currently approved therapies. Third-generation CARs combine two costimulatory domains, although added clinical benefit remains unproven. Fourth-generation CARs (‘TRUCKs’) introduce inducible cytokine modules (e.g., IL-12, IL-18) that are frequently regulated through NFAT-responsive promoters, enabling context-dependent cytokine release and tumour microenvironment modulation. Fifth-generation CARs integrate cytokine receptor–associated signalling elements, such as IL-2Rβ domains, to enhance proliferation, survival and persistence through JAK–STAT signalling pathways. **B.** Structural and functional mechanisms of major classes of armoured CAR-T cells. TRUCKs (T cells Redirected for Universal Cytokine Killing) employ inducible cytokine expression systems that are typically regulated by activation-dependent promoters following CAR stimulation. Antigen-induced CAR signalling triggers an NFAT-mediated pathway, activating an inducible promoter to drive specific responses. Cytokine-modulating CAR-T cells enhance or reshape cytokine signalling by overexpressing cytokine receptors on the cell membrane. These receptors bind cytokines within the tumour microenvironment, thereby improving CAR-T responsiveness and persistence. Antibody-secreting CAR-T cells constitutively release therapeutic antibodies or antibody-like proteins, enabling them not only to kill tumour cells but also to act as local antibody-producing cells that strengthen immune activity. **C.** Advanced CAR-T engineering strategies. Chimeric switch receptors such as PD-1/CD28 convert inhibitory ligand binding (e.g., PD-L1) into activation signalling via CD28, allowing CAR-T cells to transform tumour-derived suppressive cues into stimulatory responses and thereby overcome tumour-induced immunosuppression. Universal CAR-T cells, derived from healthy donor T cells, use interchangeable adaptors to target different tumour antigens. Gene-editing strategies are typically employed to knock out the T-cell receptor (TCR), thereby eliminating the risk of graft-versus-host disease (GvHD) and enabling off-the-shelf applicability. Switchable CAR-T systems physically separate scFv and intracellular signalling domains (CD3ζ with costimulatory modules), allowing conditional activation through small-molecule–induced heterodimerization. In the presence of the drug, the domains assemble and CAR signalling is activated; in its absence, the CAR-T cell remains inactive. CD3ζ, CD3 zeta chain; IL-2Rβ, interleukin-2 receptor beta chain; PD-1, programmed cell death 1; PD-L1, programmed death-ligand 1; scFv, single-chain variable fragment. Figure 1. Long description.

CAR-T therapy has evolved through progressive refinements in receptor design aimed at improving T-cell activation, persistence and anti-tumour efficacy. At its core, the chimeric antigen receptor (CAR) is a modular synthetic receptor that couples antibody-like antigen recognition to T-cell activation machinery (Ref. 4). It features an extracellular single-chain variable fragment (scFv) domain for specific antigen binding (e.g., CD19), a hinge domain that provides structural flexibility and influences antigen accessibility, a transmembrane domain and an internal signalling tail capable of triggering T-cell activation. Building on this, CAR-T therapy has advanced through a cycle of iterative bioengineering, with each generation designed to surmount the limitations of the last (Figure 1A) (Ref. 5). First-generation constructs (about 1990s) contained only the CD3**ζ** signalling domain, the primary activation switch (Signal 1) of the T-cell receptor. Consequently, these cells demonstrated potent *in vitro* cytotoxicity but failed to proliferate or persist *in vivo.* Second-generation constructs represent the clinical breakthrough. Scientists, recognizing that T cells require a costimulatory signal (Signal 2) for full activation and survival, added one intracellular costimulatory domain to the CD3**ζ** chain. This construct design represents the current clinical standard of care and includes all U.S. Food and Drug Administration (FDA)-approved products (Table 1) (Ref. 6). The CD28 domain, utilized in therapies like Yescarta and Tecartus, drives rapid, robust expansion and potent effector function, though this intense activity is often coupled with accelerated T-cell exhaustion. Conversely, the 4-1BB (CD137) domain found in Kymriah, Abecma and Carvykti promotes improved persistence and a long-lived central memory phenotype, resulting in a slower but more sustained proliferation profile (Ref. 7). Third-generation constructs represent a theoretical evolution, integrating two distinct costimulatory domains (e.g., CD28 and 4-1BB) to synergize activation and persistence; however, their clinical superiority over second-generation models remains a subject of ongoing investigation. Fourth-generation constructs (Figure 1B), commonly referred to as armoured CARs or T-cells Redirected for Universal Cytokine Killing (TRUCKs), represent a sophisticated advancement in therapeutic bioengineering (Ref. 8). Activated TRUCKs simultaneously lyse target cells and release pro-inflammatory cytokines (e.g., IL-12, IL-15, IL-18) to modulate the immunosuppressive tumour microenvironment (TME), thereby recruiting host immunity and rendering cold tumours susceptible to attack. Upon antigen recognition, TRUCK CAR-T cells become activated through CAR signalling. This activation triggers calcium–calcineurin–dependent NFAT signalling, which induces transcription of pro-inflammatory cytokines (e.g., IL-12, IL-15, IL-18). The localized release of these cytokines reshapes the TME, recruits host immune cells and renders cold tumours susceptible to immune attack. Fifth-generation constructs also known as armoured CARs 2.0 (Figure 1C) are engineered to express receptors for essential T-cell survival cytokines (e.g., IL-7, IL-15) or their downstream signalling components (Refs 9, 10). This design generates autonomous survival signals that sustain a stem-cell-like memory phenotype and mitigate functional exhaustion.

**Table 1.** FDA and EMA-approved CD-19-directed CAR-T cell therapies (as of 2026) Table 1. long description.

Brand name	Generic name (construct)	Target antigen(s)	Costimulatory domain	Key approved indications (relapsed/refractory)	References
Kymriah	tisagenlecleucel (tisa-cel)	CD19	4–1BB	B-cell acute lymphoblastic leukaemia (ALL) (peds/young adult); large B-cell lymphoma (LBCL); follicular lymphoma (FL)	(6, 16, 103)
Yescarta	axicabtagene ciloleucel (axi-cel)	CD19	CD28	LBCL (incl. 2L+); FL (2L+)	(6, 17)
Tecartus	brexucabtagene autoleucel (brexu-cel)	CD19	CD28	Mantle cell lymphoma (MCL); B-cell ALL (adult)	(6, 18)
Breyanzi	lisocabtagene maraleucel (liso-cel)	CD19	4–1BB	LBCL (incl. 2L+); Chronic lymphocytic leukaemia (CLL)/small lymphocytic lymphoma (SLL); MCL; FL	(6, 19)
Aucatzyl	obecabtagene autoleucel (obe-cel)	CD19	4–1BB	B-cell ALL (adult)	(20, 21)
Abecma	idecabtagene vicleucel (ide-cel)	BCMA	4–1BB	Multiple myeloma (MM) (approved for 2L+ in 2024)	(6, 22, 84)
Carvykti	ciltacabtagene autoleucel (cilta-cel)	BCMA	4–1BB	MM (approved for 2L+, ‘first as early as first relapse’)	(6, 23, 84)

*Note:* This table synthesizes data on the primary, commercially approved CAR-T products in the United States and Europe.

Importantly, CAR evolution is no longer limited to the addition of external signalling modules. Beyond combining costimulatory domains, recent studies have begun to directly redesign the core CD3ζ signalling architecture. Although CD3ζ has traditionally functioned as the primary activation module of CAR constructs, emerging evidence suggests that its structure can be modified to modulate downstream signalling strength, persistence and exhaustion profiles. Experimental work has shown that alternative CD3ζ configurations can reshape intracellular signalling programmes and enhance functional durability in preclinical models (Ref. 11). Rather than relying solely on external costimulatory domains such as CD28 or 4-1BB, this strategy focuses on re-engineering the internal activation logic of the receptor.

In addition to these intracellular and functional modifications, the extracellular antigen-binding domain of CARs is also evolving. Most approved CAR-T therapies rely on conventional scFv-based binding domains. However, alternative formats such as single-domain antibodies (nanobodies or Variable Heavy domain of Heavy chain (VHH) fragments) are now being explored. These smaller binding domains may improve stability and allow better tumour penetration, particularly in solid tumours. Preclinical studies have shown promising anti-tumour activity of VHH-based CAR constructs in solid tumour models (Ref. 12). Although clinical data remain limited, early-phase studies in hematologic malignancies (e.g., NCT04447573) suggest that VHH-based CAR-T therapy is feasible. Together, these findings indicate that next-generation CAR design involves innovation not only in intracellular signalling but also in the structure of the antigen-recognition domain. Therefore, effective CAR-T therapies must combine strong cytotoxic activity with sustained persistence, the ability to reshape the tumour microenvironment, and optimized antigen-recognition structures to overcome resistance and achieve durable clinical responses.

### Perspective of the CAR-T study

The study argues that CAR-T therapy’s demonstrated that curative potential in hard-to-treat blood cancers reflects its progression into a more advanced stage of evolution. The field’s current state is defined by a fundamental shift away from early proof-of-concept efforts and towards addressing a more complex set of next-generation challenges. Key priorities are managing long-term side effects, preventing relapse via antigen escape and conquering the barriers posed by solid tumours. To meet these clinical demands, the research landscape has shifted towards advanced synthetic biology, focusing on the engineering of intelligent and off-the-shelf (allogeneic) systems.

## The current clinical landscape: FDA and EMA-approved CAR-T therapies

By evaluating individual therapies, we can see how regulatory milestones have catalysed refinements in target selection and architecture. This product-level perspective allows us to interpret regulatory success as a metric of technical evolution rather than a series of disconnected authorizations.

### Overview of approved CAR-T products

**Figure 2.**
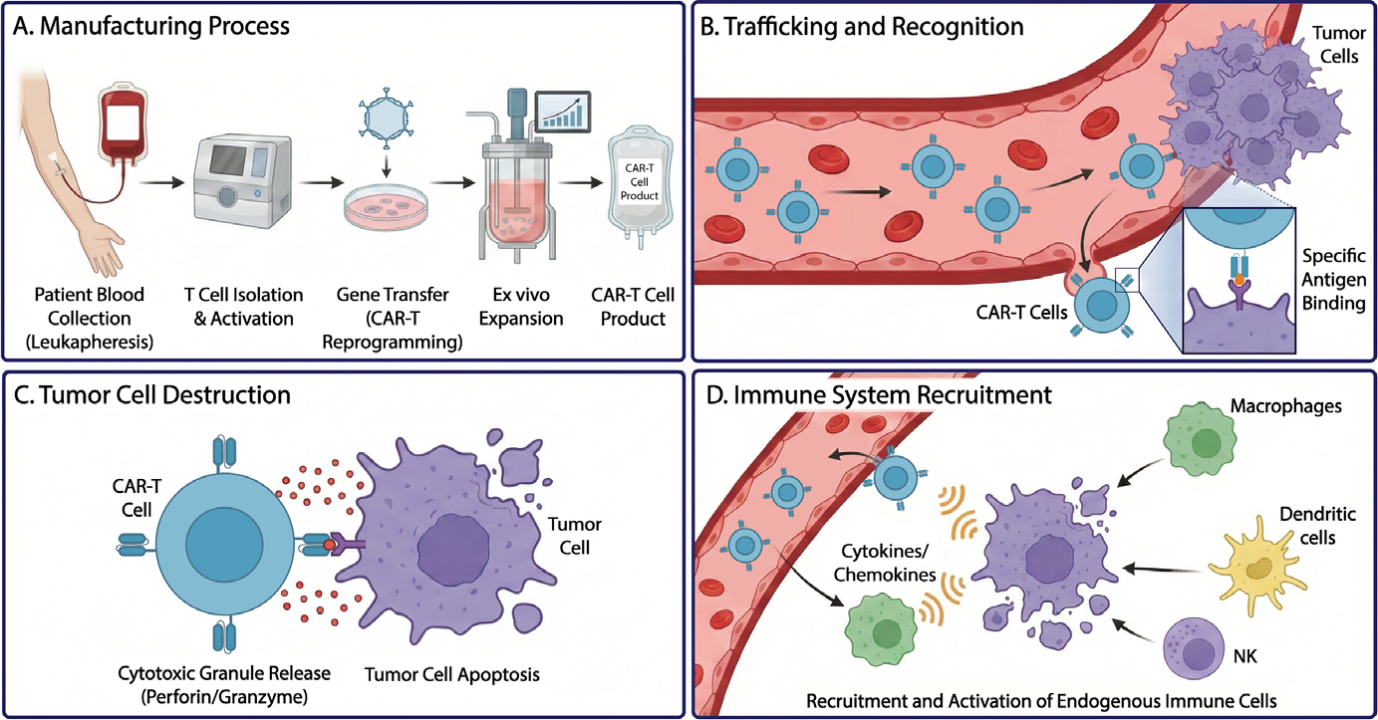
An overview of the principal phases of CAR-T cell therapy (5). The manufacturing process begins with leukapheresis to collect Peripheral Blood Mononuclear Cells (PBMCs), followed by T-cell isolation and activation. Activated T cells are genetically modified using viral or non-viral platforms to introduce the CAR transgene and expanded under controlled *ex vivo* conditions to ensure potency and viability before autologous infusion (Panel A). After administration, CAR-T cells circulate systemically towards the tumour microenvironment, where CAR-mediated antigen recognition induces immunological synapse formation and downstream activation signalling through CD3ζ and costimulatory domains (Panel B). This activation drives cytolytic activity via perforin–granzyme-mediated apoptosis and pro-inflammatory cytokine secretion, resulting in targeted tumour cell destruction (Panel C). The inflammatory milieu and release of tumour-associated antigens recruit and activate natural killer (NK) cells, macrophages and dendritic cells. This promotes secondary anti-tumour immune responses to eliminate residual tumour cells (Panel D). Figure 2. long description.

These therapies represent a cell-based gene therapy platform where a patient’s T cells are engineered to attack cancer (Figure 2). As of 2025, the clinical landscape for CAR-T therapy is dominated by a class of autologous, second-generation products approved by FDA and, in most cases, the European Medicines Agency (EMA) (Table 1) (Ref. 13). They have become a standard of care for specific relapsed/refractory (r/r) hematologic malignancies (Ref. 14). The seven major FDA-approved therapies target two primary surface antigens: CD19 for B-cell malignancies and B-Cell Maturation Antigen (BCMA) for multiple myeloma (Ref. 15).

The majority of approved products are CD19-directed therapies, designed to attach to the CD19 antigen found on certain leukaemia or lymphoma cells (Ref. 6). Kymriah (tisagenlecleucel), which utilizes a 4-1BB costimulatory domain, was the first CAR-T therapy to receive FDA approval in August 2017. Its indications include paediatric and young adult patients (up to age 25) with r/r B-cell Acute Lymphoblastic Leukaemia (ALL) and adult patients with r/r Large B-cell Lymphoma (LBCL) or r/r Follicular Lymphoma (FL) (Ref. 16). Yescarta (axicabtagene ciloleucel) employs a CD28 costimulatory domain and is indicated for adult patients with r/r LBCL and r/r FL (Ref. 17). Tecartus (brexucabtagene autoleucel), also a CD28-based construct, is approved for adult patients with r/r Mantle Cell Lymphoma (MCL) and adult r/r B-cell precursor ALL (Ref. 18). Breyanzi (lisocabtagene maraleucel) is a 4-1BB construct with a broad range of approvals for adult r/r LBCL, r/r FL (an expansion from Q2 2024), r/r Chronic Lymphocytic Leukaemia (CLL) and r/r MCL (Ref. 19). The most recent CD19-directed therapy is Aucatzyl (obecabtagene autoleucel), which was approved by the FDA in November 2024 for adult r/r B-cell precursor ALL (Ref. 20). The EMA’s Committee for Advanced Therapies (CAT) subsequently adopted a positive opinion for Aucatzyl in May 2025 (Ref. 21).

For the treatment of multiple myeloma, two 4-1BB-based constructs targeting BCMA are approved. Abecma (idecabtagene vicleucel), initially approved for late-line r/r multiple myeloma after four or more prior lines of therapy, had its approval expanded by the FDA in 2024 to patients after just two or more prior lines (Ref. 22). Carvykti (ciltacabtagene autoleucel), first approved in February 2022 for late-line use, received significant approval expansion in April 2024 (Ref. 23). This made it the first and only BCMA-targeted therapy approved for patients with r/r multiple myeloma as early as the first relapse (Ref. 6). Taken together, the current regulatory landscape reflects the clinical development of CAR-T therapy as well as its remaining limitations. Most approved CAR-T therapies are autologous, second-generation products that target only a small set of antigens, mainly CD19 and BCMA (Ref. 15). This shows that long-lasting clinical benefit has mainly come from improving the original CAR designs, rather than from developing completely new types of CAR receptors. In parallel, the absence of approved third- or later-generation CAR-T products, together with the limited efficacy observed in solid tumours, highlights ongoing challenges (Ref. 24). These include antigen escape, complex and resource-intensive manufacturing processes and insufficient long-term persistence of CAR-T cells *in vivo.* These regulatory patterns and clinical constraints support the need for next-generation strategies aimed at improving durability, scalability and expanding clinical applicability beyond hematologic malignancies.

### The evolving regulatory landscape of CAR-T

Recent regulatory changes highlight that while acute risks are now manageable, long-term safety remains poorly understood.

#### FDA REMS elimination as evidence of acute safety mastery

Effective June 26, 2025, the U.S. FDA eliminated the Risk Evaluation and Mitigation Strategy (REMS) requirement across the entire portfolio of approved CAR-T products (Ref. 25). The REMS programme, defined by its strict requirements for site certification and monitoring, was a cornerstone of early adoption but was discontinued following the determination that these specific mandates had become obsolete. The American Society for Transplantation and Cellular Therapy (ASTCT) had advocated for this change, arguing that the requirements were burdensome and duplicative, and that the clinical community’s accumulated experience over nearly a decade meant that the safe administration of these therapies and the expert management of their acute toxicities (CRS and ICANS) had become the standard of care. The REMS removal is expected to significantly lower barriers to patient access, reduce the administrative and financial burden on hospitals and encourage more treatment centres to offer CAR-T therapy, thereby addressing the significant gap between the number of eligible patients and the number of patients who receive treatment.

#### EMA PRAC warning signals the emergence of long-term risk

While the U.S. FDA’s removal of the REMS requirement signals increased confidence in managing acute toxicities, the EMA has pivoted towards stringent long-term safety surveillance within its Risk Management Plan (RMP). In June 2024, EMA Pharmacovigilance Risk Assessment Committee (PRAC) issued a major warning after identifying a new, serious risk: secondary malignancies of T-cell origin (Ref. 26). This is distinct from the patient’s original cancer. The committee reviewed 38 cases (among ~42,500 patients) of secondary T-cell lymphoma and leukaemia in patients who had received CAR-T therapy. Most critically, in seven of the cases where samples could be tested, the CAR construct itself was found to be present in the new malignant T cells, strongly suggesting a causal link where the gene therapy itself may, in rare instances, be oncogenic. Consequently, the EMA mandated that all patients treated with Abecma, Breyanzi, Carvykti, Kymriah, Tecartus and Yescarta must be monitored life-long for new malignancies.

This finding is not a contradiction of the REMS removal, but rather its logical consequence. Success in managing acute toxicities has allowed patients to survive long enough to face chronic, long-term risks. As a result, the EMA has mandated a crucial change to the product information for all CAR-T therapies, requiring that patients now be monitored life-long for the development of new, secondary malignancies. In parallel, a new CAR T-Cell Toxicities Consortium is being formed in 2025 to formally catalogue and understand these long-term adverse events.

Multiple biological mechanisms have been proposed to explain the emergence of secondary T-cell malignancies after CAR-T treatment. One proposed mechanism is insertional mutagenesis. In this scenario, integration of viral vectors near proto-oncogenes or growth-regulatory genes can disrupt normal transcriptional control (Ref. 27). Although modern self-inactivating lentiviral vectors are considerably safer than early γ-retroviral systems, they are not entirely risk-free. Their tendency to integrate into transcriptionally active chromatin may still carry a low-frequency oncogenic potential (Ref. 28). Beyond vector-related risks, intrinsic T-cell biology may also play a role. Following infusion, engineered T cells are exposed to proliferative and pro-survival signalling driven by tonic CAR activation, homeostatic cytokines and persistent antigen engagement (Ref. 29). Over time, this prolonged stimulation may promote clonal expansion and facilitate the accumulation of secondary genomic alterations (Refs 30, 31).

These mechanisms also have direct implications for next-generation CAR-T platforms, including allogeneic and CRISPR-edited products. Allogeneic CAR-T therapies may face additional selective pressure from the host, which could favour the outgrowth of rare clones with immune-evasive or proliferative advantages (Ref. 32). Similarly, CRISPR-edited CAR-T cells introduce additional genomic considerations, including double-strand DNA breaks and chromosomal rearrangements. These alterations raise concerns about long-term genomic stability. Accordingly, these potential risks necessitate long-term clinical follow-up and safety assessments tailored to each specific CAR-T platform.

Looking ahead, these safety considerations are expected to shape the next generation of CAR-T design strategies. To reduce potential risks, several approaches are being explored, including non-integrating or site-specific gene delivery methods, inducible safety switches and CAR constructs optimized to minimize tonic signalling (Refs 33, 34). Together, these strategies reflect a broader shift towards safer CAR-T therapies.

## Clinical challenges and mechanisms of failure in hematologic malignancies

The profound success of CAR-T therapy has been tempered by its unique and potent toxicities, as well as the emergence of biological resistance. The mastery of these challenges defines the current practice of cellular immunotherapy.

### Management of acute toxicities associated with CRS and ICANS

The two signature, life-threatening toxicities of CAR-T therapy are Cytokine Release Syndrome (CRS) and Immune Effector Cell-Associated Neurotoxicity Syndrome (ICANS). Both are on-target toxicities, resulting from the intended and massive activation of the engineered T cells (Ref. 35). CRS is a systemic inflammatory response caused by the massive, rapid release of pro-inflammatory cytokines notably IL-1, IL-6 and IFN-γ from both the activated CAR-T cells and, crucially, bystander myeloid cells (e.g., macrophages) that are activated by the CAR-T cells (Ref. 36). CRS typically presents within the first 1–14 days and is characterized by a flu-like syndrome (fever, malaise) that can rapidly progress to severe hypotension, hypoxia requiring mechanical ventilation and multi-organ failure. ICANS is a distinct, non-CRS-driven toxicity characterized by a toxic encephalopathic state. The mechanism is thought to involve endothelial activation and breakdown of the blood–brain barrier, allowing cytokines and T cells to enter the central nervous system. It can occur with or after CRS. Symptoms are graded based on the 10-point Immune Effector Cell-Associated Encephalopathy (ICE) score and clinical presentation, ranging from mild confusion, tremor and dysgraphia (Grade 1) to global aphasia, seizures, cerebral oedema and coma (Grade 4) (Ref. 37). Experience gained from the ASTCT’s formalized standards for toxicity grading and management ultimately facilitated REMS removal (Table 2) (Ref. 38). Although tocilizumab effectively neutralizes the IL-6 surge driving CRS, its inability to penetrate the blood–brain barrier precludes efficacy in the management of ICANS. Consequently, systemic corticosteroids remain the standard of care for ICANS. In refractory cases unresponsive to steroid intervention, emerging recent protocols prioritize the blockade of alternative inflammatory pathways, most notably via the IL-1 receptor antagonist anakinra.

**Table 2.** Simplified ASTCT consensus grading and management of CRS and ICANS Table 2. long description.

Toxicity	Grade 1 (mild)	Grade 2 (moderate)	Grade 3 (severe)	Grade 4 (life-threatening)	References
CRS	**Definition:** Fever only	**Definition:** Fever + Hypotension (no pressors) or Hypoxia (low-flow nasal cannula)	**Definition:** Fever + Hypotension (single pressor) or Hypoxia (high-flow/BiPAP)	**Definition:** Fever + Hypotension (multiple pressors) or Hypoxia (intubation)	(35, 36, 38)
	**Management:** Supportive care (e.g., antipyretics, fluids)	**Management:** Tocilizumab (anti-*IL–6R* mAb). Consider corticosteroids.	**Management:** Tocilizumab + Corticosteroids (e.g., Dexamethasone)	**Management:** Tocilizumab + High-dose Corticosteroids **+** ITU care.	
ICANS	**Definition:** ICE scores 7–9. Mild confusion, tremor, or dysgraphia	**Definition:** ICE scores 3–6. Aphasia, impaired consciousness (somnolent)	**Definition:** ICE scores 0–2. Global aphasia, stuporous, or focal seizure	**Definition:** ICE score 0. Coma, continuous seizure, or cerebral oedema	(36, 37, 38)
	**Management:** Supportive care. Increase monitoring frequency.	**Management:** Corticosteroids (e.g., dexamethasone)	**Management:** Corticosteroids (higher dose/frequency).	**Management:** High-dose Corticosteroids + ITU care.	

Abbreviations: BiPAP: biphasic positive airway pressure; CRS: cytokine release syndrome; ICANS: immune effector cell-associated neurotoxicity syndrome; ICE: immune effector cell-associated encephalopathy; IL-6R: interleukin 6 receptor; ITU: intensive therapy unit; mAb: monoclonal antibody.

Beyond their mechanistic basis, CRS and ICANS represent major clinical challenges that directly affect treatment delivery, CAR-T cell persistence and patient outcomes. Clinical studies show that toxicity profiles differ substantially between CAR constructs, particularly depending on the costimulatory domain used (Ref. 36). CD28-based CAR-T therapies are consistently associated with faster *in vivo* expansion and an earlier onset of high-grade CRS and ICANS. In contrast, 4-1BB–based constructs generally show slower expansion kinetics, delayed cytokine release and a lower incidence of severe immune-mediated toxicities (Ref. 39). These differences reflect distinct patterns of T-cell differentiation. Specifically, 4-1BB signalling favours central memory phenotypes and longer cellular persistence, whereas CD28 costimulation promotes rapid effector differentiation and earlier functional exhaustion (Ref. 40). Importantly, strategies used to manage toxicity are not biologically neutral. Early or prolonged corticosteroid use, while effective in controlling severe CRS or ICANS, has been associated with reduced CAR-T expansion and decreased durability of clinical responses in some settings (Ref. 41). Together, these findings indicate that acute toxicities are not only safety concerns but also key clinical factors that influence therapeutic efficacy, long-term disease control and the overall risk–benefit balance of CAR-T therapy.

Improvements in the management of CRS and ICANS have also influenced regulatory oversight of CAR-T therapies. Early regulatory approvals required strict REMS because of concerns about unpredictable and potentially life-threatening toxicities and the need for specialized inpatient care (Ref. 42). The development of standardized toxicity grading systems and consensus management guidelines by ASTCT improved the predictability and safety of severe immune-related toxicities. These advances reduced treatment-related mortality and the need for intensive care (Ref. 38). As clinical experience increased, and toxicity became more predictable, regulatory confidence also improved. This led to the modification or removal of REMS requirements and supported broader clinical use of CAR-T therapy, including outpatient administration in selected patient populations (Ref. 25).

### Mechanisms of relapse and treatment resistance

**Figure 3.**
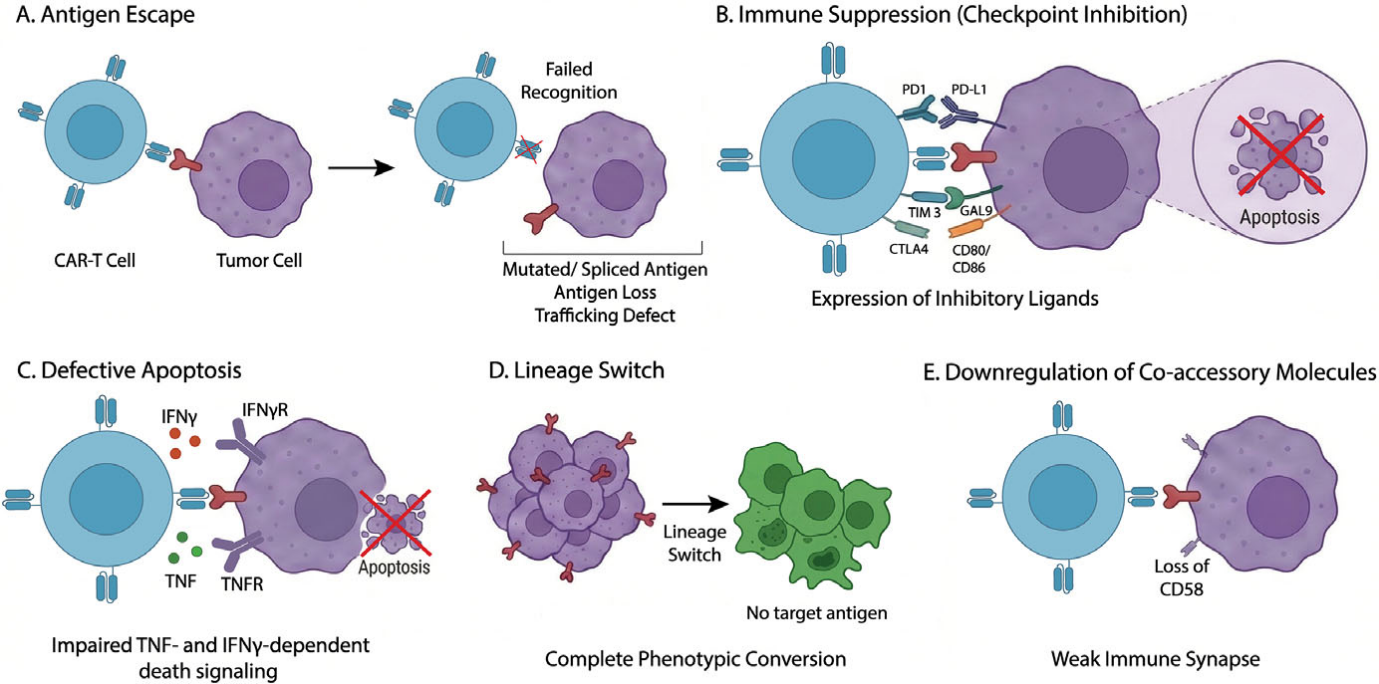
Strategies of tumour evasion from CAR-T therapy (44). **A.** Loss of the target antigen through genetic mutations, aberrant splicing, antigen masking or impaired membrane trafficking prevents CAR-mediated recognition and allows antigen-negative tumour subpopulations to expand under therapeutic pressure. **B.** Tumour cells upregulate inhibitory ligands that bind PD1, TIM3 and CTLA4 on CAR-T cells, inducing exhaustion with reduced cytokine production and impaired proliferation. **C.** Disruption of IFNγ/TNF response pathways (e.g., IFNγR or TNFR signalling defects and downstream apoptosis impairment) prevents tumour cells from undergoing apoptosis even with intact CAR-T recognition. **D.** Under selective pressure from CAR-T therapy, tumour cells may undergo lineage conversion or dedifferentiation into phenotypes lacking the targeted antigen, effectively escaping antigen-restricted CAR-T surveillance. **E.** Downregulation of accessory molecules required for optimal T-cell engagement weakens immune synapse formation, impairs CAR-T activation thresholds and reduces cytotoxic function. CTLA4, cytotoxic T lymphocyte antigen 4; GAL9, Galectin-9; IFNγ, interferon-gamma; IFNγR, IFNγ receptor; PD1, programmed cell death 1; PD-L1, programmed death-ligand 1; TIM3, T-cell immunoglobulin domain and mucin domain 3; TNF, tumour necrosis factor; TNFR, TNF receptor. Figure 3. long description.

Understanding the reasons behind CAR-T therapy failure is crucial (Figure 3). Relapses generally fall into two main categories, and distinguishing between them is a key driver of next-generation CAR-T development.

#### Antigen-positive relapse (drug failure)

In this scenario, patients relapse despite the retention of target antigen expression (e.g., CD19-positive B-ALL), indicating that therapeutic failure stems from the CAR-T product itself rather than antigen escape (Ref. 43). This outcome is primarily attributable to compromised persistence driven by T-cell exhaustion, a state of progressive dysfunction and eventual physical deletion precipitated by chronic antigen stimulation (Ref. 7). The potential for sustained therapeutic efficacy is strongly predicted by the *in vivo* persistence of CAR-T cells exhibiting stem-like or central memory characteristics (Ref. 44). In contrast, patients who relapse often have CAR-T cells that are terminally differentiated and exhausted. This outcome is linked to construct design, with CD28-based CARs driving rapid exhaustion while 4-1BB-based CARs favour memory formation.

Continuous antigen engagement leads to sustained CAR signalling, which induces transcriptional and epigenetic changes. These changes include increased expression of exhaustion-associated transcription factors such as TOX and members of the NR4A family (Refs 45, 46). In addition, stable chromatin remodelling further restricts functional recovery, even after antigen clearance (Ref. 47). This exhausted state is further reinforced by terminal effector differentiation and the loss of long-lived memory T-cell subsets, which are essential for long-term persistence and self-renewal (Ref. 48).

Clinically, these mechanisms result in limited CAR-T persistence, short-lived remissions and early relapse despite continued antigen expression. In this context, antigen-positive relapse often reflects a product-related failure rather than a resistance mechanism driven primarily by the tumour. However, antigen-positive relapse cannot be explained by T-cell exhaustion alone. Clinical and translational studies reveal substantial variability in relapse patterns driven by differences in quality of patient-derived T cells, manufacturing processes and the composition of the final CAR-T cell product (Ref. 31). Patient-specific factors, including immune competence, inflammatory state, tumour burden and prior exposure to cytotoxic therapies, also play a major role.

Together, these variables shape CAR-T expansion, persistence and functional durability, leading to divergent outcomes even among patients treated with the same CAR construct. Longitudinal clinical data further suggest that the timing of relapse provides important insight. Early relapses, typically occurring within the first few months after infusion, are more often associated with poor CAR-T expansion, limited persistence (Ref. 31). In contrast, later antigen-positive relapse may reflect progressive functional decline and loss of CAR-T persistence rather than tumour-driven resistance mechanisms. Collectively, these observations indicate that drug failure is the result of biological, technical, temporal and patient-specific factors rather than from a single dominant molecular mechanism.

#### Antigen-negative relapse (target failure)

This constitutes the more pervasive and frequent form of resistance, responsible for nearly 60% of treatment failures in CD19-targeted protocols (Ref. 43). In this scenario, the CAR-T cells are perfectly healthy and persistent, but the tumour has evolved to become invisible. This process, known as antigen escape, arises when the therapeutic pressure of CAR-T cells effectively eliminates susceptible targets, thereby selecting for resistant variants that lack the specific antigen (Ref. 43). Mechanistically, this escape is driven by a spectrum of distinct molecular pathways, as detailed in Table 3 (Ref. 49).

**Table 3.** Mechanisms of antigen escape in CD19-directed therapy Table 3. long description.

Mechanism	Description	Molecular basis	References
Alternative splicing	The tumour cell produces a non-functional, truncated version of the CD19 protein that is missing the part (epitope) that the CAR recognizes.	Mutations in splicing factors (e.g., *SRSF3*) cause the cell’s machinery to ‘skip’ exon 2 of the *CD19* mRNA. The resulting protein (*CD19*∆ex2) lacks the scFv binding site, rendering the cell invisible to the CAR-T.	(44, 52)
Lineage switching	The cancer cell reprogrammes itself, changing its entire cell type (lineage) from one that expresses CD19 to one that does not.	Most common in B-ALL with mixed-lineage leukaemia (MLL) rearrangements. Under pressure, the B-ALL (lymphoid) cell dedifferentiates and switches to an acute myeloid leukaemia (AML) (myeloid) phenotype, which does not express CD19.	(44, 53)
Clonal selection	The CAR-T therapy is 100% effective, but a tiny, pre-existing sub-clone of cancer cells that was already CD19-negative at baseline survives.	The primary B-ALL is a mix of 99.9% CD19-positive cells and 0.1% CD19-negative cells. The CAR-T perfectly kills the 99.9%, selecting for the 0.1% resistant clone, which then grows out to cause the relapse.	(4, 43, 44)
Trogocytosis	The CAR-T cell nibbles the CD19 antigen off the tumour cell surface, effectively shaving the cell and making it (and nearby cells) invisible.	A cell-to-cell interaction where the CAR-T cell physically acquires the *CD19-*membrane patch from the tumour cell. This can also lead to fratricide, where CAR-T cells now see the (stolen) CD19 on each other and kill their teammates.	(49)

Abbreviations: B-ALL: B-cell acute lymphoblastic leukaemia; scFv: single-chain variable fragment; SRSF3: serine and arginine rich splicing factor 3.

Mechanisms of antigen escape in CD19-directed therapy include alternative splicing, lineage switching, clonal selection and trogocytosis. Alternative splicing occurs when the tumour cell produces a non-functional, truncated version of the CD19 protein that is missing the part (epitope) that the CAR recognizes. Mutations in splicing factors (e.g., Serine/Arginine-Rich Splicing Factor 3 (*SRSF3*)) cause the cell’s machinery to skip exon 2 of the *CD19* mRNA. The resulting protein (*CD19*∆ex2) lacks the scFv binding site, rendering the cell invisible to the CAR-T. Lineage switching is observed when the cancer cell reprogrammes itself, changing its entire cell type (lineage) from one that expresses CD19 to one that does not. This is most common in B-ALL with Mixed-Lineage Leukaemia (MLL) rearrangements. Under pressure, the B-ALL (lymphoid) cell dedifferentiates and switches to an Acute Myeloid Leukaemia (AML) (myeloid) phenotype, which does not express CD19. Clonal selection describes a scenario where the CAR-T therapy is nearly 100% effective, but a tiny, pre-existing sub-clone of cancer cells that was already CD19-negative at baseline survives. The primary B-ALL is a mix of 99.9% CD19-positive cells and 0.1% CD19-negative cells. The CAR-T perfectly kills 99.9%, selecting for the 0.1% resistant clone, which then grows out to cause the relapse. Finally, trogocytosis involves the CAR-T cell nibbling the CD19 antigen off the tumour cell surface, effectively shaving the cell and making it invisible. This cell-to-cell interaction results in the CAR-T cell physically acquiring the CD19-membrane patch from the tumour cell, which can also lead to fratricide, as CAR-T cells acquire CD19 and become unintended targets of other CAR-T cells. This distinction between drug failure and target failure provides the central framework for guiding CAR-T development (Ref. 50).

Antigen-negative relapse represents a clinically diverse and time-dependent resistance process rather than a single, uniform molecular event. Longitudinal clinical studies show that the risk, timing and underlying mechanisms of antigen escape vary widely depending on disease subtype, baseline tumour burden and prior treatment history (Refs 31, 51). Early antigen-negative relapse is more commonly observed in patients with aggressive disease or high tumour burden at baseline. In these settings, strong selective pressure rapidly favours the outgrowth of pre-existing antigen-negative tumour clones.

In contrast, delayed antigen-negative relapse typically occurs after prolonged CAR-T persistence. This pattern reflects sustained immune pressure that gradually drives tumour adaptation through mechanisms such as alternative splicing, lineage plasticity, or clonal selection (Refs 52, 53). Importantly, clinical correlative studies indicate that specific escape mechanisms also vary according to disease context. For example, lineage switching is frequently observed in MLL-rearranged B-ALL, whereas splicing-mediated CD19 loss is more common in other lymphoid malignancies. Together, these findings suggest that antigen-negative relapse arises from both tumour-intrinsic biology and therapy-driven selection, rather than by a single conserved resistance pathway.

## The solid tumour frontier as a multifactorial barrier to CAR-T therapy

Although solid tumour resistance is often viewed as a consequence of broad biological and microenvironmental defences, these factors are more effectively analysed as a set of discrete, measurable constraints that dictate long-term therapeutic efficacy. Characterizing these barriers as dynamic and interdependent variables establishes a precise framework for addressing the performance gap currently observed between hematologic and solid malignancies.

### The challenge of CAR-T therapy in solid tumours

While CAR-T cell therapy has redefined the treatment landscape for hematologic malignancies, this success has notably failed to translate to the broader and more complex realm of solid tumours. Solid tumours (e.g., prostate, breast, lung, colon, ovarian) make up approximately 90% of all cancer cases worldwide (Ref. 54). For a decade, this resistance has been the primary focus of the field’s next-generation engineering efforts (Ref. 55). The challenge is not a single limitation but rather a sequential cascade of mechanistic barriers that impair CAR-T efficacy at multiple stages. An infused CAR-T cell must locate the tumour, infiltrate it, withstand its hostile microenvironment, recognize the cancer cells and eliminate them, all while sparing healthy tissue. Thus, solid tumours present layered obstacles that impede efficacy at every stage of the CAR-T therapy (Ref. 56).

### Three critical bottlenecks to therapeutic success

Extensive investigation has delineated three critical mechanisms that define the limitations of current therapies in solid tumours (Table 4). The first barrier is T-cell trafficking. In this scenario, CAR-T cells fail to infiltrate the tumour mass. They remain physically excluded, accumulating in circulation or peripheral stromal regions. This exclusion is driven by three distinct factors. First, physical barriers are created by a dense, fibrotic extracellular matrix (ECM) and stroma. Second, vascular barriers arise from dysfunctional, abnormal tumour vasculature that T cells cannot effectively cross. Finally, chemical barriers exist where the tumour fails to secrete the necessary homing signals (chemokines) to match T-cell receptors (Ref. 55). Recent studies show that T-cell exclusion in solid tumours is not a fixed barrier but a feature that can be modified. Engineering CAR-T cells to express matching chemokine receptors, such as CCR2b or CXCR3, or using regional delivery approaches can clearly improve tumour infiltration in preclinical models (Refs 57, 58, 59). However, better tumour infiltration alone has not led to consistent and durable clinical benefit. Although more CAR-T cells reach the tumour, their function is often limited once they arrive. Within the tumour microenvironment, CAR-T cells are exposed to suppressive signals, metabolic stress and inhibitory factors that reduce their activity and persistence (Ref. 60). Disorganized vasculature limits efficient T-cell entry and creates conditions that further suppress T-cell function (Ref. 61). As a result, even infiltrating CAR-T cells may remain poorly functional.

**Table 4.** Key barriers to CAR-T efficacy in solid tumours Table 4. long description.

Barrier	Description (the metaphor)	The mechanism of action	References
T-cell trafficking	The fortress problem	CAR-T cells fail to infiltrate the tumour. They are physically excluded, remaining in the bloodstream or trapped in the peripheral stroma. This is due to: • Physical barriers: A dense, fibrotic extracellular matrix (ECM) and stroma. • Vascular barriers: A dysfunctional, abnormal tumour vasculature that T cells cannot cross. • Chemical barriers: The tumour does not secrete the correct ‘homing’ signals (chemokines) to match the T-cell’s ‘homing’ receptors	(55, 57, 72)
Immunosuppressive tumour microenvironment (TME)	The hostile environment problem	Even if a T cell does infiltrate, it is immediately shut down by the TME. The TME is: • Metabolically hostile: Hypoxic (low oxygen) and nutrient-deprived (low glucose), forcing the T cell into a metabolic crisis. • Immunosuppressive: A soup of inhibitory factors (e.g., TGF-β, adenosine, PD-L1) and inhibitory cells (e.g., T-regulatory cells, MDSCs) designed to exhaust and deactivate T cells.	(55, 61, 66)
Antigen targeting	The friendly fire and camouflage problem	This is the most complex barrier. Even if a T cell infiltrates and survives, it must safely and effectively identify its target. • On-target, off-tumour toxicity: Unlike CD19 (only on B-cells, which are expendable), most solid tumour antigens (e.g., HER2, GPC3, CEA) are also expressed at low levels on vital healthy tissues (e.g., lungs, liver, gut). A potent CAR-T will kill the patient by attacking these healthy organs. • Antigen heterogeneity: A solid tumour is not a uniform mass. It is a camouflage of diverse cells. Some cells express Target A, some express Target B and some express none. A single-antigen CAR-T will kill some cells but leave the others (e.g., the antigen-negative cells) to survive, evolve and cause relapse.	(44, 49, 116)

Abbreviations: CEA: carcinoembryonic antigen; GPC3: glypican-3; HER2: human epidermal growth factor receptor 2; MDSCs: myeloid-derived suppressor cells; PD-L1: programmed death-ligand 1; TGF-β: transforming growth factor beta.

Together, these factors help explain why strategies that improve CAR-T trafficking in preclinical models often fail to produce durable responses in patients. Successful treatment of solid tumours will therefore require approaches that not only enhance tumour infiltration but also modify the tumour microenvironment. This may include vascular normalization strategies, such as anti-VEGF therapy, to support effective T-cell infiltration and function (Ref. 62).

The second barrier is the TME or the hostile environmental problem. Even if a T-cell successfully infiltrates the tumour, it is often functionally inhibited by metabolic stress and inhibitory signalling within the TME. This environment is metabolically hostile, being hypoxic (low oxygen) and nutrient-deprived (low glucose), which impairs T-cell function (Ref. 63). Furthermore, the landscape is profoundly immunosuppressive, defined by a dense concentration of inhibitory factors such as TGF-β, adenosine and PD-L1. These chemical signals work in concert with inhibitory cell populations, namely, regulatory T cells and Myeloid-Derived Suppressor Cells (MDSCs) to exhaust and deactivate infiltrating T cells (Ref. 56). Recent studies suggest that suppression within the TME can be actively targeted using next-generation CAR-T engineering strategies. These include dominant-negative TGF-β receptors, cytokine-secreting armoured CAR-T cells and metabolic reprogramming approaches that help restore CAR-T function under low-oxygen and nutrient-poor conditions (Refs 64, 65, 66). However, most of these approaches have shown clear benefits mainly in preclinical models. Their limited clinical impact likely reflects the complexity of the human TME, where targeting a single suppressive pathway is usually insufficient, as other pathways can maintain immune suppression.

The third and most complex barrier involves antigen targeting constraints, including on-target, off-tumour toxicity and intratumoural antigen heterogeneity. Even after infiltrating and surviving the TME, the T-cell must safely and effectively identify its target. On-target, off-tumour toxicity represents a major limitation; unlike CD19 (found only on B-cells, which are expendable), most solid tumour antigens (e.g., HER2, GPC3, CEA) are also expressed at low levels on vital healthy tissues like the lungs, liver, or gut. A potent CAR-T cell attacking these healthy organs can be fatal. In parallel, solid tumours display strong heterogeneity in antigen expression. A solid tumour is not a uniform mass but a diverse mix of cells where some express Target A, some express Target B and some express neither. A single-antigen CAR-T cell will kill some cells but leave the antigen-negative ones to survive, evolve and cause relapse. A successful solid tumour CAR-T must, therefore, be a multi-functional product, engineered with separate solutions for each of these three barriers (Ref. 5). Recent advances in antigen targeting have changed how intratumoural heterogeneity is approached. Rather than being viewed as a biological dead end, heterogeneity is increasingly treated as an engineering challenge. Dual-targeting CARs, and logic-gated systems, allow conditional antigen recognition and improve discrimination between malignant and healthy tissues (Refs 67, 68). However, the clinical use of these systems remains limited. Increased construct complexity, manufacturing challenges and the need for careful patient selection continue to restrict broader application and raise the risk of unexpected toxicity (Refs 61, 69).

In addition to these three biological bottlenecks, clinical and trial-related factors further influence therapeutic success. Most solid tumour CAR-T trials have enrolled heavily pretreated patients with advanced disease, high tumour burden and impaired immune function. These conditions inherently restrict CAR-T cell expansion, persistence and long-term function *in vivo* (Ref. 70). Trial design also contributes to these limitations, as early-phase studies are primarily focused on safety, often rely on treatment response and frequently lack appropriate control groups (Ref. 71). Collectively, these findings demonstrate that the modest efficacy of CAR-T therapy in solid tumours is a multifaceted issue rather than the result of a single variable. Biological barriers, engineering challenges and clinical conditions all interact to restrict efficacy. Future progress will likely require strategies that simultaneously address tumour trafficking, the suppressive microenvironment, antigen safety and trial design. Therefore, more durable and consistent clinical responses in solid tumours may be achieved by combining these approaches.

### Promising advances in solid tumour targeting

After nearly a decade of mostly disappointing clinical outcomes, the field has entered a phase of cautious optimism, as recent trials are finally beginning to show encouraging results in glioblastoma and other difficult solid tumours (Ref. 72). The strongest evidence supporting this renewed hope comes from a 2024 clinical study of satri-cel (Satricabtagene autoleucel) (Ref. 73). This treatment, developed by CARsgen Therapeutics, targets Claudin 18.2 (*CLDN18.2*), a tight-junction protein frequently found in gastric cancers. In a Phase 2 trial (NCT04404595) involving patients with advanced gastric or gastro-oesophageal junction cancer who had exhausted previous treatment options, satri-cel achieved a statistically meaningful improvement in both progression-free and overall survival compared to standard chemotherapy. Although side effects were substantial consistent with many CAR-T therapies, this study stands out as one of the first robust, positive, controlled trials of a CAR-T therapy in a major solid tumour setting.

However, these promising results must be viewed through the lens of the broader clinical challenges facing CAR-T therapies in solid tumours. To date, most CAR-T clinical trials in solid tumours have not shown durable responses (Ref. 70). Many studies have reported only short-term disease control or no meaningful clinical benefit. Several early-phase trials targeting solid tumour antigens such as HER2, EGFRvIII, CEA and mesothelin demonstrated limited clinical efficacy or significant on-target toxicities, and none resulted in durable, practice-changing therapeutic approvals (Refs 74, 75, 76). These largely negative or inconclusive outcomes highlight a persistent gap between strong preclinical activity and clinical efficacy in solid tumours, despite increasingly advanced CAR-T engineering strategies. In this context, the clinical success of satri-cel should be viewed as a target-specific exception rather than evidence of broadly generalizable efficacy across solid tumour CAR-T therapies. Its activity likely reflects the unique biological accessibility of CLDN18.2, rather than a universal solution applicable to solid tumours as a class (Ref. 77).

While select trials offer glimpses of clinical potential, the majority of solid tumour CAR-T research has yet to move beyond early-phase proof-of-concept into the realm of broadly effective clinical treatment.

## Next-generation engineering for designing solutions to tumour resistance

Current research is defined by the rapid expansion of an engineering toolkit designed to address the distinct challenges of tumour access, functional exhaustion, toxicity and antigen escape. The optimal therapeutic candidate will likely emerge from a combinatorial integration of these strategies.

### Enhancing access and reducing costs via allogeneic, ready-made CAR-T products

The current autologous model is a logistical bottleneck. The vein-to-vein time from cell collection to re-infusion can take weeks to months. This process is expensive, complex and prone to manufacturing failures. For patients with aggressive lymphomas or myeloma, this wait time is a fatal flaw; many progress and die before their cells are ready. Allogeneic CAR-T platform uses T cells from healthy, pre-screened donors to create a master bank of cells. This approach enables the mass engineering, expansion and cryopreservation of therapeutic cells, yielding a standardized off-the-shelf product suitable for immediate clinical application. Such an approach is poised to overcome current logistical bottlenecks, offering a cost-effective, standardized and immediately available treatment option. However, the long-term persistence, safety profile and comparative durability of allogeneic CAR-T products relative to autologous therapies are still being evaluated in ongoing clinical studies.

The central barriers to allogeneic therapy have been Graft-versus-Host Disease (GvHD), host rejection of donor cells and the belief that these cells would be less durable and less effective than autologous ones. GvHD occurs when donor T cells recognize recipient tissues as foreign due to mismatched human leukocyte antigen (HLA) molecules. In conventional settings, intact donor T-cell receptors (TCRs) can bind to host HLA–peptide complexes and trigger systemic immune activation, leading to tissue damage (Ref. 32). To reduce this risk, modern allogeneic CAR-T products frequently incorporate gene-editing strategies to disrupt the endogenous TCR and minimize alloreactivity. Data from Caribou Biosciences’ ANTLER trial (NCT04637763), presented in November 2025, have significantly advanced the clinical profile of allogeneic therapies. In this study, the allogeneic anti-CD19 product vispa-cel (CB-010) achieved an 82% overall response rate and a 51% 12-month progression-free survival in patients with second-line LBCL (Ref. 78). The safety profile was manageable, characterized by low rates of CRS and ICANS with no reported cases of clinically significant GvHD, supporting the effective control of donor-derived alloreactivity. These outcomes are particularly notable because earlier allogeneic CAR-T products frequently demonstrated limited persistence and early disease relapse, largely due to host-mediated immune rejection (Ref. 79). The sustained activity of vispa-cel is attributed to CRISPR-enabled engineering most notably a strategic PD-1 knockout which provides a cell-intrinsic checkpoint blockade to mitigate premature exhaustion. However, as a single-arm Phase 1/2 trial, the ANTLER data represent an emerging signal rather than a definitive establishment of non-inferiority to the autologous standard. While the 12-month data indicate durability for this specific CRISPR-engineered profile, longer follow-up is essential to fully characterize the risk of late immune rejection or progressive exhaustion that has historically challenged the allogeneic class. Consequently, while these findings serve as a critical proof-of-concept for the efficacy potential of gene-edited off-the-shelf products, the confirmation of their long-term comparative durability against autologous products will require larger, randomized clinical trials.

### Combatting TME barriers and exhaustion with armoured (TRUCK) CARs

The combined burden of environmental suppression and functional exhaustion represents a critical barrier to durable remission (Ref. 44). Fourth-generation TRUCKs provide a potential solution (Ref. 5). These armoured CAR-T cells are engineered to secrete a therapeutic payload upon antigen recognition, effectively turning the T-cell into a micro-pharmacy at the tumour site (Figure 1). Notable examples include huCART19-IL18 (Penn Medicine), presented at ASCO 2024, which secretes the pro-inflammatory cytokine IL-18 (Ref. 80). In a Phase 1 trial for lymphoma patients who had already relapsed after commercial CAR-T therapy, this armoured CAR-T was able to overcome resistance and induce an 80% overall response rate. Another example is the IL-12/PD-L1 Blocker Fusion (USC/City of Hope) from a 2025 Nature Biomedical Engineering study (Ref. 81). IL-12 is one of the most powerful anti-cancer cytokines but is lethally toxic if given systemically. To solve this, researchers engineered a CAR-T to secrete a fusion protein combining IL-12 with a PD-L1 blocker. The PD-L1 blocking component acts as a homing device, binding to the high levels of PD-L1 in the TME. This tethers the IL-12 payload directly to the tumour, creating a high local concentration while preventing systemic escape. In mouse models, this construct shrank solid prostate and ovarian tumours with no systemic toxicity. Although this strategy demonstrated robust anti-tumour activity in mouse models, clinical validation in humans has not yet been established. Thus, armoured CARs currently represent a promising but still largely experimental strategy for overcoming TME-mediated resistance in solid tumours.

### Smart CARs and logic-gating as a strategy to mitigate toxicity and antigen escape

Two main problems on-target, off-tumour toxicity and antigen escape could potentially be addressed by designing smart T cells capable of sensing, processing and responding. These Boolean logic-gated CARs integrate signals from multiple antigens to execute a command (Ref. 82). The AND-gate (safety gate) means the CAR-T only activates if it sees Antigen A and Antigen B. This approach represents a pivotal strategy for widening the therapeutic window, maximizing tumour selectivity while abrogating the risk of on-target, off-tumour toxicity. For example, a solid tumour expresses antigens A and B, while a healthy lung expresses only A. A conventional CAR-T targeting A would be fatal. An AND-gate CAR-T (A+B) spares the lung and kills only the tumour. This is a critical solution for solid tumours. The OR-gate (efficacy gate) means the CAR-T activates if it sees Antigen A or Antigen B. This architecture is designed to counteract antigen escape and tumour heterogeneity. For example, a tumour has a mix of CD19+ cells and CD22+ cells. A CD19-only CAR-T would leave the CD22+ cells behind (relapse). A CD19-OR-CD22 bispecific CAR-T kills both populations, preventing relapse. The NOT-gate (inhibitory gate) means the CAR-T has an activating CAR for the tumour antigen and a separate inhibitory CAR (iCAR) for a healthy antigen (Ref. 83). This solves on-target, off-tumour toxicity. For example, it kills any cell with tumour antigen unless it also has healthy antigen. The iCAR provides an inhibitory signal that overrules activation, protecting the healthy tissue. To date, most logic-gated CAR designs have shown their benefits mainly in preclinical models or small early-stage clinical studies. Whether they can improve long-term clinical outcomes in patients is still being studied.

### Combating antigen escape via novel target discovery

The utility of clinically validated targets principally CD19 and BCMA is limited by the tumour’s ability to evade detection through antigen loss. The solution is to identify new essential targets or simultaneously target multiple antigens using an OR-gate strategy. In multiple myeloma, research has already progressed beyond BCMA, with G protein-coupled receptor class C group 5 member D (GPRC5D) emerging as the next major validated target (Ref. 84). However, most of these studies are still in Phase 1 or 2, and long-term durability comparable to BCMA-directed CAR-T therapy has not yet been shown. As of 2025, numerous clinical trials are evaluating GPRC5D-directed CARs, as well as dual-targeting CARs that simultaneously target both BCMA and GPRC5D to reduce the risk of antigen escape. Other targets include Signalling Lymphocytic Activation Molecule Family member 7 (SLAMF7) and CD38. The therapeutic viability of CLDN18.2 was substantiated by a pivotal 2024 trial published in *The Lancet*, representing a major success in solid tumour target identification (Ref. 73). Other high-priority novel targets being explored in 2025 clinical trials include GPC3 for hepatocellular carcinoma and CEA for colorectal cancer (Ref. 5). Although several of these targets have entered early clinical trials, only a few are supported by randomized studies showing a clear survival benefit.

### Alternative platforms addressing key challenges

While CAR-T cells dominate the clinical landscape, the field is concurrently developing parallel adoptive cell therapy (ACT) platforms to overcome T-cell-specific limitations. TCR-engineered T-cell (TCR-T) therapy, which uses a modified natural T-cell receptor, is being developed in parallel and faces similar formidable challenges in the solid tumour landscape (Ref. 24). Importantly, a key milestone in this field occurred in August 2024, when the FDA approved the first engineered T-cell gene therapy for a solid tumour, afamitresgene autoleucel (afami-cel, Tecelra), for synovial sarcoma (Ref. 85). Unlike CAR-T therapies, which target surface antigens, this TCR-based therapy recognizes MAGE-A4, an intracellular protein presented via MHC, thereby illustrating the broader antigen-targeting potential of alternative ACT platforms. Emerging platforms are now transposing the CAR architecture into a broader spectrum of immune cell types to address the specific biological and clinical ‘blind spots’ of T cells. A comprehensive comparative analysis of these advanced cellular immunotherapies including CAR-Natural Killer (CAR-NK), CAR-macrophages (CAR-M) and CAR-T regulatory (CAR-Treg) is detailed in Table 5, which differentiates these platforms based on clinically relevant parameters such as safety profiles, trafficking capabilities and manufacturing scalability (Ref. 86). CAR-NK cells are primarily being investigated as a solution to the scalability and safety issues inherent in autologous CAR-T therapy (Ref. 13). Unlike T cells, NK cells do not induce GvHD, allowing for an allogeneic, off-the-shelf manufacturing model that bypasses the lengthy vein-to-vein wait times that currently contribute to patient mortality. Furthermore, CAR-NK cells maintain their native germline-encoded receptors, such as NKG2D, providing a secondary mechanism for tumour recognition even if the CAR-targeted antigen is downregulated a critical advantage in preventing antigen-escape relapse (Ref. 87). Similarly, CAR-M capitalizes on the natural trafficking ability of myeloid cells to home into the TME. Beyond direct phagocytosis, the clinical value of CAR-M lies in their ability to remodel the TME, potentially reprogramming the niche from a pro-tumourigenic cold state to a pro-inflammatory hot state by recruiting and activating endogenous T cells (Ref. 88). Finally, CAR-Treg cells are being developed specifically for autoimmune applications and organ transplantation (Ref. 89). By providing localized, antigen-specific immunosuppression, CAR-Tregs aim to induce tolerance and avoid the systemic toxicity associated with traditional long-term immunosuppressive regimens.

**Table 5.** A detailed comparison of CAR-T, TCR-T, CAR-NK, CAR-M and CAR-Treg therapies Table 5. long description.

Therapy	Mechanism of action	Target specificity	Efficacy	Safety profile	Persistence and expansion	Clinical applications	Advantages and limitations	References
CAR-T	Engineered T cells expressing chimeric antigen receptors (CARs) that recognize surface antigens and directly kill target cells	Surface antigens (e.g., CD19, BCMA)	High efficacy in hematologic malignancies; some success in solid tumours	Cytokine release syndrome (CRS), neurotoxicity, risk of secondary malignancies	Long persistence in blood, robust expansion	B-cell leukaemias/lymphomas, multiple myeloma; investigational in solid tumours	Very potent; limited by antigen escape and tumour microenvironment in solid tumours	(1, 6, 111)
TCR-T	T cells engineered with T-cell receptors (TCRs) that recognize intracellular antigens presented on MHC	Peptide/MHC complexes (intracellular antigens) (e.g., NY-ESO–1, MAGE-A4)	Can target a broader set of antigens, including intracellular proteins; moderate to high efficacy	CRS less common than CAR-T; potential off-target reactivity	Moderate persistence; expansion depends on antigen and patient context	Solid tumours (melanoma, sarcoma, HPV cancers); some hematologic malignancies	Broader target repertoire; HLA-restricted, which limits patient eligibility	(24, 85)
CAR-NK	Natural killer (NK) cells expressing CARs; kill via CAR and natural cytotoxicity	Surface antigens; innate NK targets (e.g., CD19, NKG2DL)	Early clinical trials show promise in hematologic and some solid tumours	Lower CRS and neurotoxicity risk; generally safer than CAR-T	Shorter persistence than CAR-T; may require multiple doses	Hematologic cancers, some solid tumours	Off-the-shelf potential; safer, but limited durability and expansion	(86, 121)
CAR-M	Macrophages engineered with CARs to phagocytose tumour cells and modulate tumour microenvironment	Surface antigens (e.g., CD19, HER2)	Early-stage clinical evidence; preclinical models show tumour clearance	Low CRS; inflammatory responses possible	Persistence variable; primarily acts locally	Solid tumours, especially immunosuppressive tumours	Can infiltrate solid tumours better than CAR-T; still experimental with limited clinical data	(86, 121)
CAR-Treg	Regulatory T cells engineered with CARs to suppress immune responses	Surface antigens on target tissues (e.g., CD19, HLA-A2)	Mainly studied in autoimmune diseases and transplantation; efficacy in controlling immune responses	Risk of immunosuppression; generally safe	Moderate persistence; needs antigen presence to survive	Autoimmune diseases, graft-versus-host disease, transplant rejection	Suppresses unwanted immune activity; not for cancer therapy; still experimental	(86, 123)

Abbreviations: CAR-NK: CAR-natural killer cells; CAR-M: CAR-macrophages; CAR-Treg: CAR-regulatory T cells; HER2: human epidermal growth factor receptor 2; HLA: human leukocyte antigen; HLA-A2: human leukocyte antigen A2; HPV: human papillomavirus; MAGE-A4: melanoma-associated antigen 4; MHC: major histocompatibility complex; NKG2DL: natural killer group 2, member D ligand; NY-ESO-1: New York oesophageal squamous cell carcinoma 1; TCR-T: T-cell receptor T-cell therapy.

## Advancing CAR-T through automation and long-term considerations

Biological hurdles such as immunosuppression, T-cell exhaustion, toxicity and antigen heterogeneity have influenced both the architecture of CAR-T cells and the practicality of their large-scale delivery. As CAR-T therapies grow more intricate and bespoke, logistical hurdles such as limited manufacturing capacity and lack of standardization have emerged as decisive factors that govern their ultimate clinical success.

### Emerging technologies in manufacturing and delivery

One of the most significant barriers to CAR-T access is not biological, but logistical. The current manufacturing paradigm a centralized, manual, craft-brewed process is the bottleneck (Ref. 90). In 2019, an estimated 450,000 patients were eligible for CAR-T; this is expected to hit 2 million by 2029. In 2021, the entire global infrastructure produced only 4,000 doses with reported capacity differing across regions (Ref. 91). This represents a major limitation in manufacturing scale. Available reports suggest that, in certain healthcare settings, approximately 25% of eligible multiple myeloma patients may experience mortality while awaiting access to a manufacturing slot, with outcomes varying across regions (Refs 92, 93). Automating and decentralizing appear to be the answer. The field is moving towards closed, automated, end-to-end manufacturing platforms capable of performing isolation, activation, transduction and expansion with minimal human intervention. Cellmation is one example of a digital platform intended to connect and control modular instruments across this workflow (Ref. 94). The integration of automated systems may reduce operator-dependent variability, lower the cost of goods and improve product standardization. Crucially, this enables point-of-care manufacturing, empowering hospitals to utilize automated benchtop platforms for on-site production.

Although several closed and automated systems are already used in commercial CAR-T manufacturing, point-of-care production remains limited. It is currently confined to pilot programmes in a small number of academic and high-volume centres. Strict regulatory requirements, including GMP compliance, batch release testing, consistency between sites and quality control, continue to restrict broader clinical implementation. For these reasons, decentralized and hospital-based manufacturing should be viewed as emerging and centre-dependent approaches, rather than fully established alternatives to centralized production models (Ref. 95).

In addition to automation and decentralization efforts, a more radical strategy under investigation is *in vivo* CAR engineering. While current innovations aim to optimize the existing *ex vivo* manufacturing workflow, *in vivo* programming seeks to bypass it entirely. Instead of isolating and genetically modifying T cells outside the body, CAR-encoding genetic material is delivered directly to T cells within the patient using targeted viral vectors or lipid nanoparticle systems. Preclinical studies have demonstrated that functional CAR-T cells can be generated *in vivo*, leading to effective tumour control in animal models (Refs 96, 97, 98). This approach has the potential to shorten treatment timelines, reduce infrastructure demands and improve scalability. However, challenges related to targeting specificity, dose control and long-term genomic safety remain under investigation (Ref. 95). Although still experimental, *in vivo* CAR programming represents a conceptual shift in how CAR-T therapies may be manufactured and delivered in the future.

### The application of computational biology in CAR design

The adoption of artificial intelligence (AI) and machine learning is increasingly being explored to augment specific phases of the CAR-T workflow, ranging from early-stage construct screening to retrospective patient stratification. While AI is moving into the realm of computer-based prediction and optimization, these applications remain largely in the developmental phase. In the design phase, computational frameworks are being utilized as high-throughput screening tools to identify CAR architectures with favourable biophysical properties. Deep learning models have been applied to analyse and refine new CAR constructs *in silico*, with the goal of optimizing for attributes such as binding affinity and tonic signalling before laboratory synthesis (Ref. 99). For example, recent computational pipelines have successfully guided the refinement of tandem bi-specific CARs to improve surface expression and anti-tumour potency in preclinical models (Ref. 100). However, these *in silico* evolved constructs have yet to demonstrate superior clinical outcomes in human trials compared to traditionally designed receptors.

AI-driven patient stratification currently represents a translational research frontier rather than a routine clinical tool. Machine learning models are being trained on historical clinical and imaging data to identify patterns associated with therapeutic outcomes (Ref. 101). Toxicity prediction models can analyse baseline patient data (e.g., tumour burden, C-reactive protein, ferritin) to identify correlations with severe CRS or ICANS. While these models offer a potential framework for earlier risk identification, they currently function as experimental decision-support prototypes that lack the prospective validation required for routine clinical management.

The potential for efficacy prediction is exemplified by retrospective analyses of AI-based models, such as those applied to pre-treatment PET/CT scans from the JULIET trial (NCT02445248). These models identified radiomic signatures associated with poor clinical outcomes (Refs 102, 103), suggesting a future where clinicians might use such data to stratify patients towards more potent interventions, such as armoured CAR constructs. Nevertheless, the transition towards ultra-precision oncology where models forecast individual failure mechanisms like antigen escape faces significant hurdles. These include a scarcity of high-quality, standardized clinical datasets for model training and the necessity of explainable AI frameworks to meet evolving regulatory requirements for drug manufacturing and clinical decision-making.

Overall, while AI-integrated approaches for CAR design and patient selection are promising, they are currently limited to preclinical proof-of-concept studies or retrospective clinical analyses. Prospective validation and randomized evidence are essential to establish these technologies as reliable tools. Consequently, these systems should be regarded as emerging research methodologies rather than established drivers of CAR-T product design or routine clinical practice.

### Current clinical landscape and future perspectives

Recent scientific meetings have highlighted continued progress in CAR-T therapy, particularly in oncology. At American Society of Hematology (ASH) 2024, new data were presented on dual-targeting CAR constructs and strategies for difficult-to-treat diseases such as acute myeloid leukaemia (AML) (Refs 104, 105). In AML, targets including CD33, CD123 and CLL1 are under active clinical evaluation. Some studies are testing dual-targeting combinations or inhibitory CAR designs to improve safety while maintaining efficacy. Similarly, American Association for Cancer Research (AACR) 2025 keynote presentations focused on efforts to enhance CAR-T activity in solid tumours through advanced engineering strategies (Ref. 106).

Beyond oncology, CAR-T therapy is being explored in selected B-cell–driven autoimmune diseases. Early clinical studies have reported treatment-free remissions in severe refractory conditions such as lupus (Ref. 107), myasthenia gravis (Ref. 108) and multiple sclerosis (Ref. 109). However, these trials remain early-phase and involve limited patient numbers. Larger controlled studies are needed before these approaches can be considered established therapies. Applications in regenerative medicine are at an even earlier stage. iPSC-based strategies, including programmes targeting diseases such as type 1 diabetes, are largely experimental and remain conceptual at present (Ref. 110). These directions represent long-term research efforts rather than near-term clinical applications.

Despite this expansion into new therapeutic areas, oncology remains the most mature and evidence-supported domain of CAR-T therapy. Long-term follow-up data show that CD19-directed CAR-T can induce durable remissions and may be curative in a subset of patients with B-cell malignancies (Refs 111, 112). In multiple myeloma, BCMA-targeted CAR-T therapies similarly achieve long-term responses and extended treatment-free intervals. Despite these advances, broader clinical implementation remains challenging. Regulatory requirements, manufacturing constraints and high costs continue to limit wider adoption, particularly outside specialized centres (Ref. 95). Expanding access will require improvements in regulatory coordination, manufacturing standardization, infrastructure and integration into health-care systems.

### Market dynamics and patent trends in CAR-T therapy

The clinical development of CAR-T therapy has been accompanied by rapid commercial expansion, with increasing patent activity in CAR-T and TCR-T, particularly in the United States and China (Ref. 24). Collaboration between academia and industry has played a central role in scaling next-generation platforms, including off-the-shelf products and approaches for solid tumours (Ref. 113).

Despite this commercial momentum, broader clinical adoption remains constrained by manufacturing standardization, regulatory complexity and cost. Automated and decentralized systems must still satisfy GMP requirements, validated process controls, release criteria and inter-site consistency (Refs 95, 114). In parallel, individualized production, specialized infrastructure and intensive monitoring continue to drive high costs, while real-world cost-effectiveness and reimbursement pathways remain variable across health-care systems (Ref. 115). Improved coordination between regulation, manufacturing standards and health-economic planning will be necessary to ensure sustainable and equitable access to CAR-T therapies.

## Navigating emerging challenges and the engineering revolution to establish curative CAR-T therapy in modern oncology

As of 2026, CAR-T has progressed from experimental last resort to standard-of-care, curative-intent therapy for selective patient subsets with B-cell malignancies and multiple myeloma. The 2025 elimination of the REMS requirement marks a critical inflection point, signalling that the risks of CRS and ICANS are now manageable enough to allow for streamlined, broader patient access. Moreover, more than 10 years of follow-up data show that, for a subset of patients, especially those with well-defined CD19-positive B-cell malignancies, CD19-directed CAR-T can be curative, providing durable, treatment-free remissions.

Paradoxically, the clinical triumph of CAR-T has unmasked a new set of intricate challenges, shifting the research focus from initial efficacy to long-term management and optimization. Today, CAR-T therapy must overcome both the problem of treatment resistance and the formidable barrier presented by solid tumours (Ref. 116). In hematologic malignancies, resistance is primarily driven by antigen escape, where tumours evade detection through mechanisms such as alternative splicing or lineage switching (Ref. 43). Solid tumours pose a distinct set of challenges, manifesting as a sequential series of barriers ranging from deficient trafficking and stromal exclusion to local immunosuppression and the risks of off-tumour toxicity (Ref. 13). Attention has shifted from acute toxicity management to the identification of chronic, long-term risks. The June 2024 EMA warning concerning secondary T-cell malignancies has redefined safety protocols, emphasizing the critical necessity of life-long patient monitoring (Ref. 26). Finally, the inherent lack of scalability within the autologous model has created a substantial bottleneck, hindering both commercial viability and broad clinical adoption. Operational inefficiencies and long vein-to-vein times compromise the therapeutic potential, preventing delivery to a majority of eligible patients, a significant fraction of whom face mortality while on the waiting list (Ref. 91).

The future of CAR-T therapy is being shaped by a rapid expansion of synthetic biology, bioengineering and computational science, all converging to address the major challenges that have emerged from the field’s early successes. CAR-T is no longer envisioned as a simple killer cell but as a multi-functional, intelligent therapeutic platform (Ref. 24). By 2030, the therapies most likely to become standard of care in selected indications will be those designed to tackle, all at once, the logistical, biological and safety barriers that now restrict CAR-T technology.

Next-generation designs increasingly rely on allogeneic, off-the-shelf cells created through CRISPR editing to eliminate alloreactivity (Ref. 117). This strategy is now substantiated by encouraging 2025 clinical data demonstrating efficacy comparable to autologous products in defined clinical settings (Ref. 118). To overcome the solid tumour barrier, these constructs are being armoured through fourth-generation TRUCK designs capable of secreting transgenic cytokines such as IL-12 or IL-18, thereby converting immunologically cold tumours into inflamed-hot ones (Ref. 119). At the same time, safety and durability are being enhanced through smart, logic-gated architectures that use Boolean systems such as AND-gates for precise tumour recognition or OR-gates to counter antigen heterogeneity to minimize toxicity and prevent escape (Ref. 120).

Advances in manufacturing and computation are serving to accelerate this engineering transformation (Ref. 121). Automated, decentralized point-of-care production platforms are emerging as solutions to the long-standing access bottleneck, while AI is accelerating every stage of development from *in silico* CAR design to machine-learning models that predict toxicity and efficacy before treatment (Ref. 101).

## Conclusion

In conclusion, CAR-T therapy has firmly established itself as a curative cornerstone in hematologic oncology. The field has entered a dynamic second phase, marked by vigorous, multidisciplinary innovation. The integration of synthetic biology, allogeneic platforms and computational science is actively addressing the complex challenges of resistance, toxicity and accessibility. The field is expanding beyond traditional CAR-T approaches, incorporating TCR-T cells to target intracellular antigens, CAR-NK cells for safer, GvHD-free allogeneic therapy, and CAR-macrophages that actively infiltrate and remodel the tumour microenvironment (Ref. 122). This therapeutic paradigm is expanding beyond oncology, with compelling 2025 data demonstrating deep remissions in autoimmune diseases and a promising trajectory towards regenerative medicine (Ref. 123). Supported by compelling 2025 data in solid tumours and rapid clinical expansion, the engineered CAR-T therapy platform is poised to overcome existing barriers and define the next era of 21st-century medicine (Ref. 124).

## Data Availability

This study did not generate or analyse any datasets, and data sharing is not applicable.

## Search databases and inclusion criteria

The literature for this review was identified through a comprehensive search of PubMed, MEDLINE, Google Scholar and ClinicalTrials.gov for studies published through February 2026. Additional data were sourced from the FDA, EMA, pharmaceutical manufacturers and industry news outlets. The search used terms including, but not limited to: ‘CAR-T cell therapy’, ‘chimeric antigen receptor’, ‘adoptive immunotherapy’, ‘hematological malignancies’ and ‘solid tumours’. Articles were selected based on the following inclusion criteria: primary research and clinical trial results (Phases I–III); high-impact systematic reviews and meta-analyses; studies involving FDA/EMA-approved CAR-T products (e.g., Tisagenlecleucel, Axicabtagene ciloleucel); and English-language publications in peer-reviewed journals.

## Figure 1. Long description

Panel A at the top displays five horizontally arranged CAR constructs, each with labeled extracellular, transmembrane, and intracellular regions. From left to right: First Generation CAR shows a single-chain variable fragment (scFv), linker, hinge, and CD3ζ chain. Second Generation adds one co-stimulatory molecule. Third Generation includes two co-stimulatory molecules. Fourth Generation introduces a cytokine encoding gene regulated by NFAT, with cytokine release indicated. Fifth Generation incorporates an IL-2 receptor beta domain and JAK/STAT signaling elements. Panel B in the middle row shows three armoured CAR-T mechanisms. Left: TRUCK CAR-T depicts inducible cytokine release (e.g., IL-12) via NFAT-driven gene expression. Center: Cytokine Modulating CAR-T illustrates overexpression of cytokine receptors, enhancing response to environmental cytokines. Right: Antibody-secreting CAR-T shows continuous secretion of antibody-like proteins from an encoded gene. Panel C at the bottom presents three advanced strategies. Left: Chimeric Switch CAR-T features a PD-1/ CD28 switch receptor converting inhibitory signals to activation. Center: Universal CAR-T shows gene editing to remove the T cell receptor, enabling off-the-shelf use. Right: On-switch CAR-T separates signaling domains, activated only in the presence of a small molecule, toggling between ‘off’ and ‘on’ states.

Navigate back to Figure 1..

## Table 1. Long description

The table contains seven rows of therapies and six columns. The header columns, from left to right, are Brand name, Generic name (construct), Target antigen(s), Costimulatory domain, Key approved indications (relapsed or refractory), and References. The first row lists Kymriah with tisagenlecleucel (tisa-cel), targeting CD19, using 4-1BB, indicated for B-cell acute lymphoblastic leukaemia in pediatric or young adult patients, large B-cell lymphoma, and follicular lymphoma, with references 6, 16, 103. The second row is Yescarta with axicabtagene ciloleucel (axi-cel), targeting CD19, using CD28, for large B-cell lymphoma including second line or later, and follicular lymphoma second line or later, references 6, 17. The third row is Tecartus with brexucabtagene autoleucel (brexu-cel), targeting CD19, using CD28, for mantle cell lymphoma and adult B-cell acute lymphoblastic leukaemia, references 6, 18. The fourth row is Breyanzi with lisocabtagene maraleucel (liso-cel), targeting CD19, using 4-1BB, for large B-cell lymphoma including second line or later, chronic lymphocytic leukaemia or small lymphocytic lymphoma, mantle cell lymphoma, and follicular lymphoma, references 6, 19. The fifth row is Aucatzyl with obecabtagene autoleucel (obe-cel), targeting CD19, using 4-1BB, for adult B-cell acute lymphoblastic leukaemia, references 20, 21. The sixth row is Abecma with idecabtagene vicleucel (ide-cel), targeting BCMA, using 4-1BB, for multiple myeloma approved for second line or later in 2024, references 6, 22, 84. The seventh row is Carvykti with ciltacabtagene autoleucel (cilta-cel), targeting BCMA, using 4-1BB, for multiple myeloma approved for second line or later, first as early as first relapse, references 6, 23, 84.

Navigate back to Table 1..

## Figure 2. Long description

Panel A, top-left, shows the manufacturing process: from left, a blood draw labeled Patient Blood Collection (Leukapheresis), followed by a machine labeled T Cell Isolation and Activation, a petri dish labeled Gene Transfer (CAR-T Reprogramming), a bioreactor labeled *Ex vivo* Expansion, and a bag labeled CAR-T Cell Product. Panel B, top-right, illustrates trafficking and recognition: CAR-T cells move through a blood vessel toward a cluster of Tumor Cells, with an inset showing Specific Antigen Binding between a CAR-T cell and a tumor cell. Panel C, bottom-left, depicts tumor cell destruction: a CAR-T cell releases red dots labeled Cytotoxic Granule Release (Perforin/Granzyme) toward a Tumor Cell, which is undergoing apoptosis. Panel D, bottom-right, shows immune system recruitment: CAR-T cells and cytokines/chemokines recruit and activate endogenous immune cells, including Macrophages, Dendritic cells, and NK cells, to the tumor site.

Navigate back to Figure 2..

## Table 2. Long description

From left to right, the first column lists toxicity types CRS and ICANS, each spanning two rows for definition and management. The next four columns represent Grade 1 mild, Grade 2 moderate, Grade 3 severe, and Grade 4 life-threatening. For CRS, definitions progress from fever only to fever with hypotension or hypoxia, increasing in severity from no pressors or low-flow oxygen to multiple pressors or intubation. Management escalates from supportive care to tocilizumab, corticosteroids, and ITU care. For ICANS, definitions use ICE scores, with Grade 1 as ICE 7 to 9 (mild confusion, tremor, dysgraphia), Grade 2 as ICE 3 to 6 (aphasia, somnolence), Grade 3 as ICE 0 to 2 (global aphasia, stupor, focal seizure), and Grade 4 as ICE 0 (coma, continuous seizure, cerebral oedema). Management increases from supportive care and monitoring to escalating doses of corticosteroids and ITU care. The last column cites references for each toxicity. Abbreviations are explained below the table.

Navigate back to Table 2..

## Figure 3. Long description

Panel A at the top left shows a CAR-T cell encountering a tumor cell with a target antigen. An arrow points to a tumor cell lacking the antigen, labeled ‘Failed Recognition,’ with text below reading ‘Mutated/Spliced Antigen, Antigen Loss, Trafficking Defect.’ Panel B at the top right depicts a CAR-T cell and a tumor cell with surface ligands labeled PD1, PD-L1, TIM3, GAL9, CTLA4, CD80, and CD86. An arrow points to a tumor cell with a red X over an apoptotic cell, labeled ‘Apoptosis.’ The caption reads ‘Expression of Inhibitory Ligands.’ Panel C at the bottom left shows a CAR-T cell releasing IFNγ and TNF toward a tumor cell with IFNγR and TNFR. An arrow points to a tumor cell with a red X over ‘Apoptosis,’ labeled ‘Impaired TNF- and IFNγ-dependent death signaling.’ Panel D in the center bottom shows a CAR-T cell and a tumor cell, with an arrow leading to a cluster of green cells labeled ‘Lineage Switch’ and ‘No target antigen,’ with the caption ‘Complete Phenotypic Conversion.’ Panel E at the bottom right shows a CAR-T cell and a tumor cell with reduced surface molecules, labeled ‘Loss of CD58,’ and the caption ‘Weak Immune Synapse.’

Navigate back to Figure 3..

## Table 3. Long description

The first row describes alternative splicing, where tumour cells produce a truncated CD19 protein missing the CAR-recognized epitope. Mutations in splicing factors such as SRSF3 cause skipping of exon 2 in CD19 mRNA, resulting in CD19∆ex2 protein lacking the scFv binding site, making cells invisible to CAR-T. References are 44 and 52. The second row covers lineage switching, where cancer cells change lineage from CD19-expressing to non-expressing types, most common in B-ALL with MLL rearrangements. Under therapy pressure, B-ALL cells dedifferentiate to acute myeloid leukaemia (AML), which does not express CD19. References are 44 and 53. The third row details clonal selection, where CAR-T therapy eliminates CD19-positive cells but a pre-existing CD19-negative sub-clone survives and expands, causing relapse. The primary B-ALL consists of 99.9 percent CD19-positive and 0.1 percent CD19-negative cells; CAR-T selects for the resistant clone. References are 4, 43, and 44. The fourth row explains trogocytosis, where CAR-T cells physically acquire CD19-membrane patches from tumour cells, making both tumour and CAR-T cells invisible. This can lead to fratricide, as CAR-T cells attack each other upon acquiring CD19. Reference is 49. Abbreviations listed are B-ALL for B-cell acute lymphoblastic leukaemia, scFv for single-chain variable fragment, and SRSF3 for serine and arginine rich splicing factor 3.

Navigate back to Table 3..

## Table 4. Long description

The table has four columns labeled Barrier, Description (the metaphor), The mechanism of action, and References. The first row describes T-cell trafficking as the fortress problem, where CAR-T cells fail to infiltrate the tumour due to physical barriers like a dense extracellular matrix and stroma, vascular barriers from abnormal vasculature, and chemical barriers from mismatched chemokine signals. References are 55, 57, 72. The second row covers the immunosuppressive tumour microenvironment, called the hostile environment problem, where infiltrating T cells are shut down by hypoxic and nutrient-deprived conditions and immunosuppressive factors such as TGF-β, adenosine, PD-L1, T-regulatory cells, and MDSCs. References are 55, 61, 66. The third row details antigen targeting as the friendly fire and camouflage problem, highlighting on-target, off-tumour toxicity where solid tumour antigens like HER2, GPC3, and CEA are also present on healthy tissues, risking damage to organs, and antigen heterogeneity where tumour cells express different targets, allowing antigen-negative cells to survive and cause relapse. References are 44, 49, 116. Abbreviations listed include CEA for carcinoembryonic antigen, GPC3 for glypican -3, HER2 for human epidermal growth factor receptor 2, MDSCs for myeloid-derived suppressor cells, PD-L1 for programmed death-ligand 1, and TGF-β for transforming growth factor beta.

Navigate back to Table 4..

## Table 5. Long description

From the top row, the table lists therapies in the first column: CAR-T, TCR-T, CAR-NK, CAR-M, and CAR-Treg. For each therapy, the following attributes are detailed left to right. Mechanism of action: CAR-T uses engineered T cells with chimeric antigen receptors to recognize surface antigens and directly kill target cells; TCR-T uses T cells with engineered T-cell receptors recognizing intracellular antigens on MHC; CAR-NK uses natural killer cells expressing CARs for killing via CAR and innate cytotoxicity; CAR-M uses macrophages with CARs to phagocytose tumour cells and modulate the tumour microenvironment; CAR-Treg uses regulatory T cells with CARs to suppress immune responses. Target specificity: CAR-T and CAR-NK target surface antigens such as CD19 and BCMA or NKG2DL; TCR-T targets peptide/MHC complexes including NY-ESO-1 and MAGE-A4; CAR-M targets surface antigens like CD19 and HER2; CAR-Treg targets surface antigens on tissues such as CD19 and HLA-A2. Efficacy: CAR-T shows high efficacy in hematologic malignancies and some solid tumours; TCR-T can target a broader antigen set with moderate to high efficacy; CAR-NK shows promise in early trials; CAR-M has early-stage evidence and preclinical tumour clearance; CAR-Treg is mainly studied for immune control in autoimmune and transplant settings. Safety profile: CAR-T is associated with cytokine release syndrome, neurotoxicity, and secondary malignancy risk; TCR-T has less frequent cytokine release syndrome but potential off-target effects; CAR-NK has lower cytokine release syndrome and neurotoxicity risk; CAR-M has low cytokine release syndrome but possible inflammatory responses; CAR-Treg carries immunosuppression risk but is generally safe. Persistence and expansion: CAR-T persists long in blood with robust expansion; TCR-T has moderate persistence; CAR-NK persists less and may need repeat dosing; CAR-M persistence is variable and mainly local; CAR-Treg has moderate persistence requiring antigen presence. Clinical applications: CAR-T is used for B-cell leukemias/lymphomas and multiple myeloma, investigational in solid tumours; TCR-T is used for solid tumours and some hematologic malignancies; CAR-NK is for hematologic cancers and some solid tumours; CAR-M is for solid tumours, especially immunosuppressive types; CAR-Treg is for autoimmune diseases, graft-versus-host disease, and transplant rejection. Advantages and limitations: CAR-T is potent but limited by antigen escape and tumour microenvironment; TCR-T has a broader repertoire but is HLA-restricted; CAR-NK is safer and off-the-shelf but less durable; CAR-M can infiltrate solid tumours but is experimental; CAR-Treg suppresses immune activity, not for cancer, and is experimental. References are listed for each therapy in the final column. Abbreviations are defined below the table.

Navigate back to Table 5..
